# Supplementary material for: From sequence to enzyme mechanism using multi-label machine learning
Source: BMC Bioinformatics. 2014 May 19;15:150. doi: 10.1186/1471-2105-15-150 (PMC4229970; doi:10.1186/1471-2105-15-150)
Supplement: Additional file 2 — Java code of ml2db. Additional file ml2db_code.tar.gz contains the Java source code to run the multi-label machine learning experiments and save the results to database. The code’s Javadoc is included. [file 1471-2105-15-150-S2.zip › additional file 2/ml2db/utils/doc/uk/ac/standrews/utils/main/ReflectionUtils.html]

ReflectionUtils


JavaScript is disabled on your browser.


- Overview
- Package
- Class
- Use
- Tree
- Deprecated
- Index
- Help

- Prev Class
- Next Class

- Frames
- No Frames

- All Classes

- Summary:
- Nested |
- Field |
- Constr |
- Method

- Detail:
- Field |
- Constr |
- Method


uk.ac.standrews.utils.main

## Class ReflectionUtils

- java.lang.Object
- - uk.ac.standrews.utils.main.ReflectionUtils

- ---

    

  ```
  public class ReflectionUtils
  extends java.lang.Object
  ```

  Class

  Version:
  :   24 Feb 2011

  Author:
  :   Luna De Ferrari luna.deferrari-at-ed.ac.uk

- - ### Constructor Summary

    Constructors

    Constructor and Description

    `ReflectionUtils()`
  - ### Method Summary

    Methods

    | Modifier and Type | Method and Description |
    | --- | --- |
    | `static java.lang.reflect.Field[]` | `classFieldNames(java.lang.Object o)` public static String classFieldsNamesAndValuesString(Object o){ TreeMap map = ReflectionUtils.classFieldsNamesAndValues(o); String fieldsValues = "Fields of " + o.getClass().getName() + "\n"; Set> entries = map.entrySet(); Iterator> it = entries.iterator(); while(it.hasNext()){ Entry entry = it.next(); fieldsValues += entry.getKey() } return fieldsValues; } |
    | `static java.util.TreeMap<java.lang.String,java.lang.String>` | `classFieldsNamesAndValues(java.lang.Object o)` |

    - ### Methods inherited from class java.lang.Object

      `equals, getClass, hashCode, notify, notifyAll, toString, wait, wait, wait`

- - ### Constructor Detail


    - #### ReflectionUtils

      ```
      public ReflectionUtils()
      ```
  - ### Method Detail


    - #### classFieldNames

      ```
      public static java.lang.reflect.Field[] classFieldNames(java.lang.Object o)
      ```

      public static String classFieldsNamesAndValuesString(Object o){
      TreeMap map =
      ReflectionUtils.classFieldsNamesAndValues(o); String fieldsValues =
      "Fields of " + o.getClass().getName() + "\n"; Set>
      entries = map.entrySet(); Iterator> it =
      entries.iterator(); while(it.hasNext()){ Entry entry =
      it.next(); fieldsValues += entry.getKey() }
      return fieldsValues;
      }


    - #### classFieldsNamesAndValues

      ```
      public static java.util.TreeMap<java.lang.String,java.lang.String> classFieldsNamesAndValues(java.lang.Object o)
      ```


- Overview
- Package
- Class
- Use
- Tree
- Deprecated
- Index
- Help

- Prev Class
- Next Class

- Frames
- No Frames

- All Classes

- Summary:
- Nested |
- Field |
- Constr |
- Method

- Detail:
- Field |
- Constr |
- Method
